# Pulmonary Rehabilitation in Management of Chronic Obstructive Pulmonary Disease

**DOI:** 10.7759/cureus.18414

**Published:** 2021-10-01

**Authors:** Mohammed G Alharbi, Harjeevan S Kalra, Megha Suri, Nitin Soni, Nkiruka Okpaleke, Shikha Yadav, Suchitra Shah, Zafar Iqbal, Pousette Hamid

**Affiliations:** 1 Internal Medicine, California Institute of Behavioral Neurosciences & Psychology, Fairfield, USA; 2 Internal Medicine, Prince Mohammed Bin Abdulaziz Medical City, Aljouf, SAU; 3 Internal Medicine, California Institute of Behavioral Neurosciences & Psychology, Firefield, USA; 4 Pediatrics, California Institute of Behavioral Neurosciences & Psychology, Fairfield, USA; 5 Medicine, California Institute of Behavioral Neurosciences & Psychology, Fairfield, USA; 6 Psychiatry and Behavioral Sciences, California Institute of Behavioral Neurosciences & Psychology, Fairfield, USA; 7 Internal Medicine, Kathmandu University, Kathmandu, NPL; 8 Emergency Medicine, California Institute of Behavioral Neurosciences & Psychology, Fairfield, USA; 9 Emergency Medicine, The Kidney Centre, Karachi, PAK; 10 Neurology, California Institute of Behavioral Neurosciences & Psychology, Fairfield, USA

**Keywords:** lung disease, obstructive, copd, rehabilitation, exercise, training

## Abstract

Chronic obstructive pulmonary disease (COPD) is an obstructive and progressive airway disorder that is linked with a significant loss in daily physical activity as well as psychological issues that contribute to the patient's impairment and poor health-related quality of life. Over the last two decades, however, the research and application of nonpharmacologic therapies such as lung rehabilitation have been expedited with increasing evidence of systemic events in COPD patient groups and their nugatory impact on their functioning pulmonary rehabilitation (PR). It is a key part of integrated treatment for COPD and other chronic breathing disorders and is helpful in supporting the recovery of patients following COPD hospitalization. In this paper, we summarize current evidence regarding the effectiveness of PR in the management of chronic COPD. A systematic review was carried out during June 2021, searching databases PubMed, Google Scholar, and EBSCO. The authors extracted qualitative data, and then the author's names, year, study type, methodology, and the result were reported. The search of the aforementioned databases returned a total of 127 studies that were included for title, abstract, and full-text screening, and nine studies were enrolled for final data extraction.

PR alleviates tiredness and dyspnea, improves emotional function, and increases the ability to do daily activities. These benefits are relatively extensive and substantial clinically. Rehabilitation acts as an important component of COPD management and helps to improve the quality of life and training linked to health.

## Introduction and background

Chronic obstructive pulmonary disease (COPD) is an obstructive and progressive airway disorder linked with a significant loss in daily physical activity and psychological issues that contribute to the patient's impairment and poor health-related quality of life [1]. COPD is a prominent cause of death and morbidity globally, accounting for about 147,000 deaths and 700,000 hospitalizations in the United States each year. Around 15.7 million Americans have been diagnosed with COPD, but an estimated 50% of persons with COPD go misdiagnosed; thus, the true number of people affected is likely considerably higher [2].

One of the major processes causing these extrapulmonary consequences is the chronic systemic inflammation associated with COPD, which may begin or aggravate concomitant conditions such as cardiovascular disease, osteoporosis, anemia, type two diabetes, lung cancer, and depression [3]. As a result, COPD patients are disabled by the disease's systemic manifestations, with the most significant systemic dysfunction in COPD patients being peripheral muscle dysfunction caused by both physical inactivity and systemic inflammation, to which we can add undernutrition, hypoxemia, oxidative stress, and systemic corticosteroid therapy [4]. This peripheral muscle dysfunction has to do, in other words, with a decrease in early lactic acidemia and oxidative stress, muscle fiber volume, fiber types redistributions (change from type one to type two of fibers), and altered fiber capillarization [5].

COPD treatment aims to enhance the functioning and quality of life of a patient by maintaining normal lung function, reducing symptoms, and preventing exacerbations [6]. For a long time, COPD therapy has been focused mostly on improving pharmacological blockade. However, over the last two decades, the research and application of nonpharmacologic therapies such as lung rehabilitation have been expedited with increasing evidence of systemic events in COPD patient groups and their nugatory impact on their functioning (pulmonary rehabilitation [PR]) [7].

PR is defined as a "guided treatment method for restoring the function of a patient." It consists of a comprehensive intervention based on a thorough patient evaluation, followed by treatments tailored to the patient's needs, such as exercise training, education, and behavior change, all aimed at improving physical and psychological conditions and promoting long-term adherence health-improving behaviors [8].

The advantages of PR are chronic lung disease patients and can be useful for individuals regardless of the degree of their lung disease. It is a key part of integrated treatment for COPD and other chronic breathing disorders and helps support the recovery of patients following COPD hospitalization. One of the major results of PR is the survival advantage [5]. In addition, the benefits of complete PR programs to COPD patients include reducing symptoms (dyspnea and tiredness), improving exercise tolerance and health-related quality of life (HRQoL), reducing the need for health care, and increasing physical activity. In several investigations, increases in maximum exercise and functional exercise capacity, including post-lung transplantation, have been observed in patients with different chronic lung disorders [9].

PR is currently a key therapy for patients with chronic lung disease; numerous advantages have been proven, including improving activity, decreasing dyspnea, improving the quality of life associated with health, and reducing health utilization [7,9]. In this paper, we summarize current evidence regarding the effectiveness of PR in managing chronic COPD.

## Review

Methodology

Study Design

A simple qualitative systematic review was conducted following the preferred reporting items for systematic reviews and meta-analyses (PRISMA 2020) guidelines [10].

Study Duration

This review was conducted in June 2021.

Search Strategy

A systematic electronic search on PubMed, Google Scholar (Google, Mountain View, California), and EBSCO (EBSCO Information Services, Ipswich, Massachusetts) was carried out using the following terms in different combinations: lung disease, obstructive; COPD; rehabilitation; exercise; and training. Along with other keywords. We included all full texts (randomized controlled trials, systematic reviews, and prospective studies) to make up this study. The following keywords were utilized to find relevant literature, which conformed to Medical Subject Headings (MeSH) terms in PubMed or subject terms in EBSCO; " Pulmonary rehabilitation," "Chronic obstructive pulmonary disease," "COPD," and "PR." Boolean operators including "OR" and "AND" were used in combination with the appropriate keywords. The search results included full texts, freely available articles, human trials, and the English language.

Selection Criteria

Inclusion criteria: All relevant studies with similar objectives as our study. Time and language restrictions were made to 20 years and English language due to lack of translation sources.

Exclusion criteria: All studies irrelevant to our topic and papers published 20 years ago or more.

Data Extraction

The authors extracted qualitative data, and then the author's names, year, study type, methodology, and the result were reported.

Data Management

The data was extracted based on a specific form on Microsoft Excel Worksheet (Microsoft Corporation, Redmond, Washington). The authors reviewed data to determine the initial findings and the modalities of performing the surgical procedure. Each member's outcomes were revised again to minimize the mistakes and ensure the data's validity.

Risk of Bias Assessment

The Newcastle-Ottawa scale (NOS) was utilized for qualitative and quantitative data synthesis for the prospective control and clinical studies, the Cochrane Collaboration in the Cochrane Handbook for Systematic Reviews of Interventions and contained in RevMan, and the AMSTAR checklist for systematic reviews and meta-analyses were used to evaluate the included research quality [11-13]. The reviewers investigated and addressed any conflicts in the quality evaluation.

Strategy for Data Synthesis

Summary tables, including the collected details from the relevant studies, were produced to provide a qualitative overview of the included study characteristics and result data. The extent of the recommended pooled analysis was investigated once the data processing was evaluated. After completing the data extraction in this meta-analysis, decisions were taken on how to better use case and control data. Regardless of the validity of the pooled meta-analyses, a qualitative synthesis of the determined data was performed to summarize current evidence regarding the effectiveness of PR in the management of chronic COPD.

Results

Search Results

**Figure 1:**
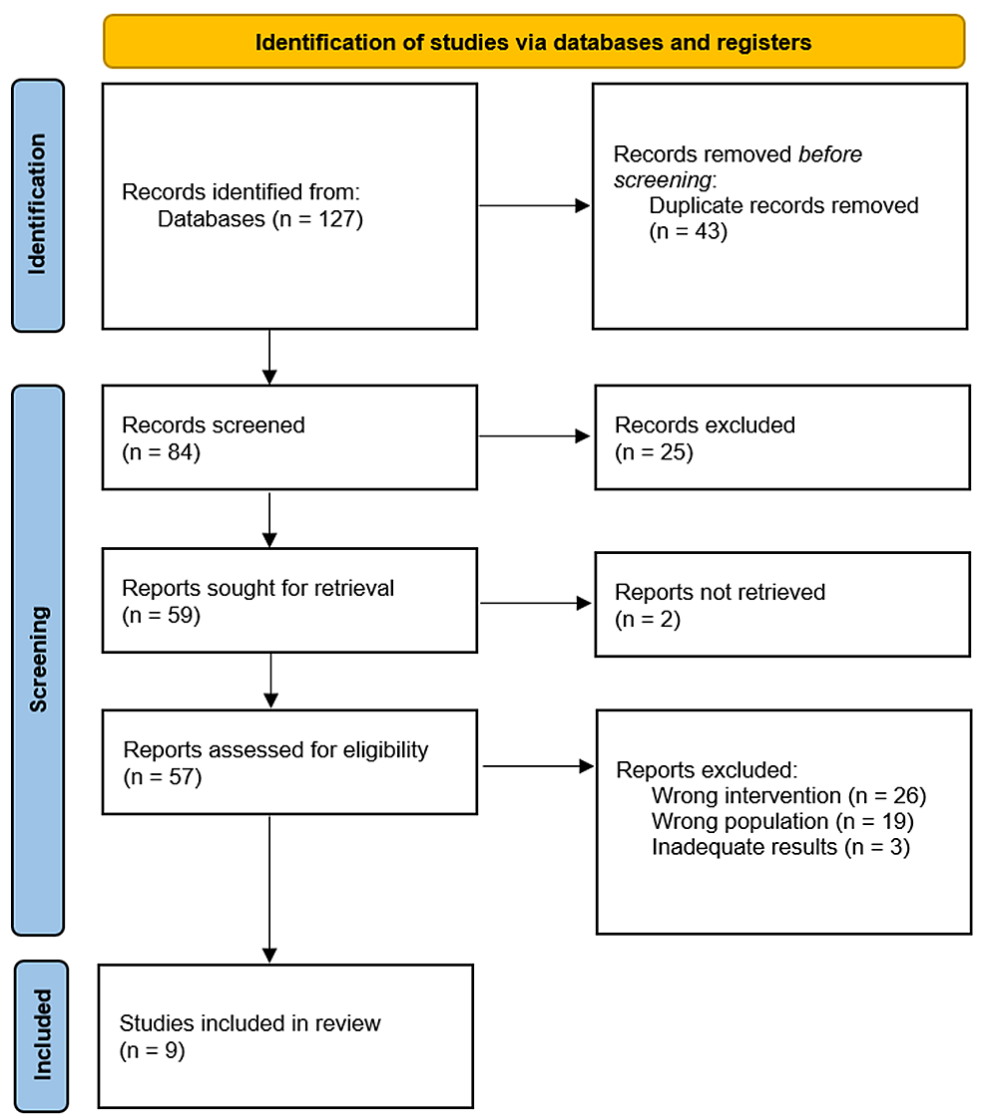
PRISMA flow chart for the systematic search process PRISMA - preferred reporting items for systematic reviews and meta-analyses

The search of the mentioned databases returned a total of 127 studies that were included for title screening. Eighty-four of which were enrolled for abstract screening, by which 25 articles were excluded. The remaining 59 publications were enrolled for full-text assessment. The full-text revision led to the exclusion of 32 studies, and only nine studies were enrolled for final data extraction (Figure 1).

Qualitative Data Synthesis

A total of nine studies were included in the qualitative data synthesis of this review, six of which are trials, and three are systematic reviews and meta-analyses [11-19].

Using the St. Respiratory George's questionnaire (SGRQ), the 6-minutes walking test (6-MWT), and the BODE index as the primary outcome measures, Barakat et al. evaluated an entirely outpatient-based PR program in patients with chronic obstructive pulmonary disease COPD and found significant changes within the components of the SGRQ (12.3 for the score total) for the patients of COPD [11].

Salman et al. discovered that rehabilitation groups in 20 trials (979 patients) performed considerably better on the walking test than control groups. The Chronic Respiratory Disease Questionnaire was used in 12 studies (723 patients), and the rehabilitation groups experienced less shortness of breath than the control groups. Only respiratory muscle training was utilized in some studies. Still, trials that included at least lower-extremity training showed that rehabilitation groups did substantially better than control groups on the walking test and shortness of breath. Only when rehabilitation programs lasted six months or longer did rehabilitation groups outperform control groups in trials involving severe COPD patients. In trials including mild/moderate COPD patients, rehabilitation groups outperformed control groups in short- and long-term rehabilitation programs [12].

Xu et al. studied the effects of modified PR on patients with moderate to severe COPD. The PR group got conventional therapy, nursing, and modified PR for 12 weeks. In contrast, the control group received conventional treatment, pursed-lip breathing, nursing, and abdominal breathing training for 12 weeks. The two groups were compared in terms of baseline characteristics, the SGRQ, the 6MWT, the modified medical research council (MMRC) dyspnea scale, and lung function. The PR group significantly improved the 6MWT (p=0.05), while the control group had no statistically significant differences. MMRC was significantly lower in the PR group than in the control group. The patients' pulmonary function in both groups remained constant [13].

Wilson et al. assigned participants to one of two groups: maintenance or standard care. Every three months for a year, the maintenance program consisted of two hours (one hour of individually tailored exercise instruction and one hour of education). The change in Chronic Respiratory Questionnaire (CRQ) dyspnea score (primary end goal) at 12 months, which was 0.19 (0.26 to 0.64) units, and other CRQ domains showed no significant differences between the groups. There were no differences in body fat, EQ5D, MET-minutes, activity rating, Hospital Anxiety and Depression Scale (HADS), exacerbations, or hospitalizations [14].

Katajisto et al. reported that 75 of the patients had completed the course of PR, and 92% had shown substantial clinical improvement. When compared to the previous year, their hospital days were cut by 54%. One year following the course, 53% of patients indicated that they had remained with regular exercise training [15].

In a randomized controlled trial of 150 patients hospitalized with a COPD exacerbation, Kjærgaard et al. found that two months of PR resulted in a considerably better improvement in the pulmonary function than the same rehabilitation program started two months after discharge and that two months of PR resulted in a better overall improvement in the pulmonary function. The difference in the endurance shuttle walk test was borderline significant, while the COPD assessment test yielded no significant results [16].

Rubí et al. tested the effectiveness of this PR program by comparing health resource use from the year before and the year after PR. Clinical variables including dyspnea, the BODE index, and the Chronic Respiratory Questionnaire, and health resources use, including the number of exacerbations, the number of hospitalizations, and days of hospitalization. Significant improvements after PR were found in dyspnea, exercise capacity, and quality of life and on the BODE index. Compared with the 12 months before PR, there were also significant reductions in exacerbations, hospitalizations, and days of hospitalization [17].

Puhan et al. have indicated that current trials have shown no effect in-hospital rehabilitation and mortality and have shown a heterogeneity relative to the most recent update. The comprehensive rehabilitation programs and the methodological quality of the research included can explain in some way the variability of effects on hospital readmissions and death [18].

McCarthy et al. found statistically significant improvement for all included outcomes. In four important domains of quality of life (QoL) CRQ scores for dyspnoea, fatigue, emotional function, and mastery, the effect was larger than the minimal clinically important difference (MCID) of 0.5 units. The SGRQ showed statistically significant improvements in all domains, with a total score improvement of more than four units. Both functional and maximal exercise improved in a statistically meaningful way. Researchers found that those who received PR had a higher maximum exercise capacity (mean Wmax [W]) than those who received standard treatment [19]. Study characteristics are presented in Table 1.

 

**Table 1:** Study characteristics: author, year, country, study type, methods, and outcomes (n=9) COPD - chronic obstructive pulmonary disease, PR - pulmonary rehabilitation, RCT - randomized controlled trial

Author, year, country	Study type	Method	Outcomes
Barakat et al. (2008) [11]	A prospective, parallel-group controlled study	Study of an outpatient rehabilitation program in 80 patients with COPD. The active group (n = 40) took part in a 14-week rehabilitation program (3 h/wk, 1.5 h of education and exercise, and 1.5 h of cycling). The control group (n = 40) was reviewed routinely as medical outpatients.	The rehabilitation program, without any change in pulmonary function, considerably improved the quality of life and exercise tolerance of moderate COPD patients and the risk of mortality in the rehabilitated patients was also significantly lower when assessed by the BODE index
Salma et al. (2003) [12]	Systematic review and meta-analysis	MEDLINE, Cinhal, and Cochrane Library searches for trials of rehabilitation for COPD patients. The search identified 69 trials, of which 20 trials were included in the final analysis.	The COPD patients who are rehabilitated have higher training capabilities and less breathability than the ones who are not rehabilitated. The short- and long-term rehabilitation of patients with mild or moderate COPD is beneficial, whereas the rehabilitation program of patients with severe COPD is at least 6 months in length.
Xu et al. (2017) [13]	A randomized controlled trial	A total of 125 participants were recruited in this study (sixty-five in the PR group and sixty-two in the control group). The PR group was treated in 12 weeks of conventional therapy, nursing, and modified lung rehabilitation, whereas the control group had conventional, nursing, breathing, and abdominal respiratory training for the 12 weeks.	Modified PR decreases dyspnea symptoms, boosts training capacity, and enhances the quality of life of moderately to severe COPD patients.
Wilson et al. (2015) [14]	A randomized controlled trial	148 patients with COPD who had completed at least 60% of a standard PR program. Patients were randomized to receive a maintenance program or standard care. The maintenance program consisted of two hours (one hour individually tailored exercise training and one hour education program) every three months for one year.	A three-monthly two-hour maintenance program in COPD patients after 12 months did not enhance results.
Katajisto et al. (2017) [15]	Clinical trial	Seventy-eight inactive patients with severe COPD were recruited for a PR course; three of them did not finish the course. The course took 6-8 weeks and included 11-16 supervised exercise sessions.	PR is efficient when measured by saved hospital days in severe COPD. Half of the patients could be motivated to continue exercising on their own.
Kjærgaard et al. 2020 [16]	A randomized controlled trial	150 patients hospitalized with an exacerbation of COPD, participants were allocated to pulmonary rehabilitation either within two weeks after discharge or the same rehabilitation program but initiated two months after discharge.	After an acute exacerbation of COPD, early pulmonary rehabilitation led to a quicker improvement in physical performance compared with later in a stable phase but did not enhance survival or prolonged periods of inpatient rehabilitation.
Rubí et al. (2010) [17]	A prospective research trial	72 patients who underwent PR program completed the PR intensive phase	A multidisciplinary, outpatient PR program substantially reduces health resource use in patients with severe and very severe COPD.
Puhan et al. (2016) [18]	Systematic review and meta-analysis	The study identified studies through searches of the Cochrane Central Register of Controlled Trials (CENTRAL), MEDLINE, Embase, PEDro (Physiotherapy Evidence Database), and the Cochrane Airways Review Group Register of Trials.	Evidence shows moderate to large effects of rehabilitation on health-related quality of life and exercise capacity in patients with COPD after an exacerbation.
McCarthy et al. (2015) [19]	Systematic review and meta-analysis	The study identified 65 RCTs involving 3822 participants from the Cochrane Airways Group Specialized Register.	Pulmonary rehabilitation reduces dyspnea and tiredness, boosts emotional function, and improves the sensation of control of people. These gains are medium-sized and substantial clinically. Rehabilitation is an important part of COPD administration and helps to improve the quality of life linked to health and capacity for practice.

Quality Assessment

The risk of bias in the prospective clinical trials was conducted using NOS and presented in Table 1. Risk of bias assessment of the included systematic reviews and meta-analyses was conducted and presented in Table 2. Only one study fulfilled all the AMSTAR scale requirements, indicating a low risk of bias and a high level of evidence [18].

**Table 2:** Quality assessment checklist for systematic reviews (AMSTAR)

AMSTAR questions	Salman et al. (2003) [12]	Puhan et al. (2016) [18]	McCarthy et al. (2015) [19]
Was an 'a priori' design provided?	Yes	Yes	No
Was there duplicate study selection and data extraction?	Yes	Yes	Yes
Was a comprehensive literature search performed?	Yes	Yes	Yes
Was the status of publication (i.e. grey literature) used as an inclusion criterion?	No	Yes	Yes
Were the characteristics of the included studies provided?	Yes	Yes	Yes
Was the scientific quality of the included studies assessed and documented?	Yes	Yes	Yes
. Was the scientific quality of the included studies used appropriately in formulating conclusions?	Yes	Yes	Yes
Were the methods used to combine the findings of studies appropriate?	No	Yes	Yes
Was the likelihood of publication bias assessed?	Yes	Yes	Yes
Was the conflict of interest included?	No	Yes	No

Risk of Bias of the Included Randomized Control Trials

The nature of the intervention meant that it would not blind participants and professionals who provided the sessions. The risk of performance bias was therefore high in all investigations. The risk of bias in various areas of bias varies among the studies included, and in some included studies, inadequate detail has been supplied to inform judgment.

**Figure 2:**
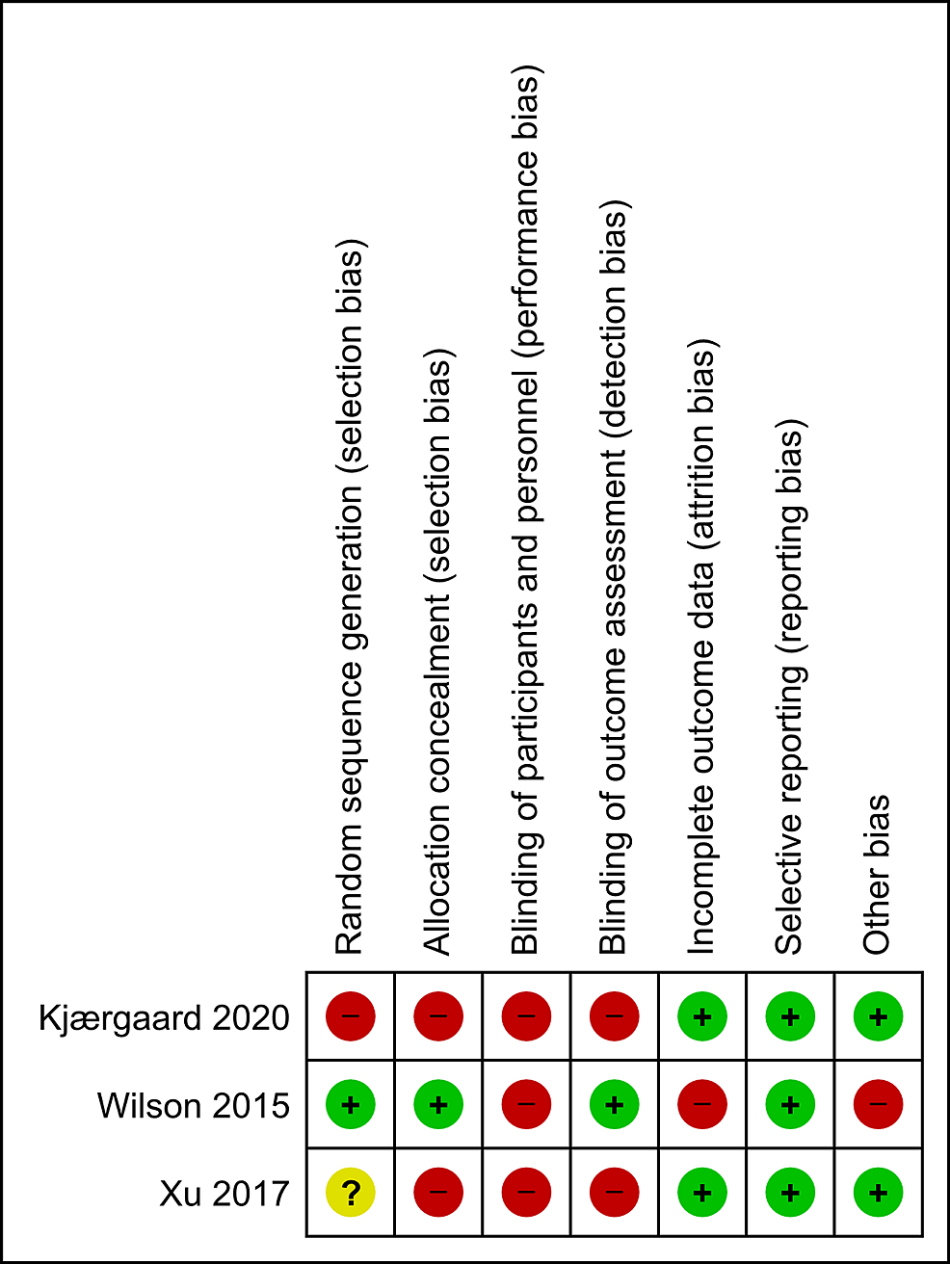
Risk of bias summary for each included study

**Figure 3:**
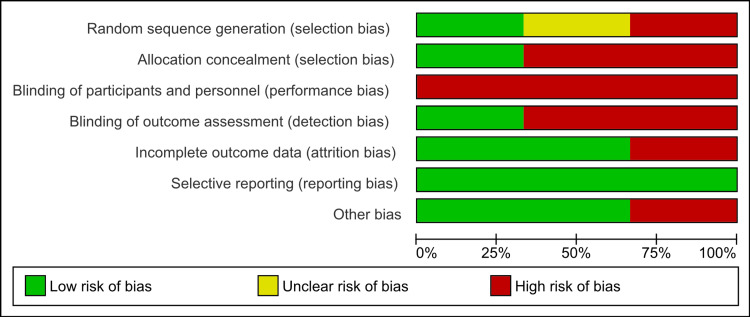
Risk of bias as percentages in included studies

Discussion

PR offers advantages for all patients with chronic respiratory diseases of any kind who, despite adequate pharmaceutical therapy, suffer from a decline in their pulmonary function, are symptomatic, and have sensitivity to stress [20]. A comprehensive assessment of the patient's lung condition and current severity should be done before the commencement of the PR program. The duration of the COPD patients' symptoms, present symptoms, MMRc scoring, history of smoking, testing of pulmonary function, blood gas analysis, oxygen supplementation, and noninvasive ventilation are all included. Special care should be made to the comorbidities of the patient. This is crucial since numerous additional medical issues might affect the course and ability of the patient to exercise. These may include obesity, obstructive sleep apnea (OSA), diabetes, cardiovascular disorders, hypertension, osteoarthritis, lung hypertension, peripheral and pulmonary vascular disease [6].

A PR program's evaluation of patients and program results is a critical component. Patients' conditions, including symptoms, endurance and strength, health-related quality of life, and so on, should be measured by rehabilitation therapists prior to training. We should evaluate patient performance and program efficacy before, during, and after a period of training [10]. Exercise training is the foundation of PR and encompasses a wide range of activities, including ground walking, cycling, strength training, water-based exercise training, Tai Chi, and so on. The best kind of training for COPD patients is determined by their physiologic needs and individual demands [14]. Different published papers have assessed the effects of PR programs on COPD patients through multiple evidentiary assessments. The efficacy and usefulness of PR were shown. The major results were given, including exercise results, dyspnea, HRQoL, psychological benefits, cost efficiency, decreased use of health care, and survival [11- 13,15- 17,19].

Griffiths et al. reported the outpatient program, administered during six weeks and followed up at 12 months. The rehabilitation group randomly modified both the exercise ability and the health quality of life significantly. The authors showed the economic health advantages connected with the completion of the rehabilitation program of particular importance [21]. Previous Lacasse et al. meta-analysis has shown that COPD rehabilitation increases workout capability and quality of existence, including patients with shortness of breath [22]. PR also leads to significant health expense reductions. Previous studies by Lertzman et al. and Jensen also demonstrated a reduction in hospitalizing and shorter hospital stay resulting in PR [23,24]. Nine centers with 647 patients have recently gained a multi-center and community-based experience that has consistently shown benefits of dyspnoea and quality of life and reduced the use of healthcare services (for example, doctor visits, phone calls, hospital days, urgent care) in 18 months [25].

Xu et al. observed substantial improvements in the exercise tolerance of 6-MWT and the level of dyspnea assessed by MMRC following 12 weeks of modified PR [13]. After twelve weeks of practice, Cheng et al. noticed substantial increases in maximum performance two times a week [26]. It has been established by Altenburg et al. that four factors are associated with improving the endurance exercise capacity in COPD patients after seven weeks of exercise [27]. Participants changed their tidal and respiratory volumes considerably in three additional high-intensity trials (70%-80% maximal activity) [28-30]. Sundararajan et al. investigated the specific effect of a 6-week outpatient PR program and found an improvement in walking distance, dyspnea score, and health status [31].

It is assumed that the specific specified exercise education program will evoke physiological adjustments indicated in the peak-oxygen uptake measurements (VO2). A series of research has gathered and reported high VO2 levels, showing improvements in this cardiorespiratory fitness standard gold measurement [32, 33]. A recent article, which brings together data from several rehabilitation centers, reported significant increases in high volume VO2 and is likely one of the largest groups of its kind [34].

The major obstacles are lack of motivation or incompatibility, mental disease, untreated cardiovascular disabilities, incapacity to practice for orthopedic reasons or other reasons, as well as unstable illnesses (e.g., hepatic disease, diabetes) [35]. Although current smokers have the same PR advantages, smokers usually adhere inadequately to PR than former smokers. Contraindications to PR are not absolute contraindications. Before PR, patients are urged to stop smoking [6]. Few pieces of research found the predicted variables of non-compliance with PR. There is, however, social isolation, [36] depression, and decreased quadriceps strength, in addition to active smoking [37]. The results of a review published in 2014 were that compliance with PR was approximately the same as compliance with medicine, i.e., 50% [38]. A retrospective study has furthermore shown that the risk of COPD patients completing a PR program is less when they are present smokers, attend a prolonged program, have experienced frequent exacerbations requiring hospital admission in the previous year, have a long journey to the center, and have a high Medical Research Council (MRC) level of dyspnea [39]. In a more recent study, Keating et al. found travel and transportation as obstacles to completion of a PR program and a lack of perceived benefit of PR, being a current smoker, sickness, and depression [40].

## Conclusions

The results of this review back up the importance of PR in the treatment of COPD patients. Their role is relatively extensive and substantial clinically. Rehabilitation acts as an important component of COPD management and helps to improve the quality of life and training linked to health. In COPD patients, rehabilitation increases walking ability and shortness of breath. Rehabilitation regimens may assist all individuals with symptoms of COPD. Patients with severe COPD may benefit from a 6-month rehabilitation program, but those with mild to moderate COPD may benefit from shorter programs. These findings shed fresh light on the efficacy of COPD rehabilitation. They may help guide future studies and determine which PR components are crucial, their optimal length and location, supervision, and training intensity.
